# American Dental Association

**Published:** 1871-09

**Authors:** 


### AMERICAN DENTAL ASSOCIATION.

This association met at the appointed place—White Sulphur Springs—on Tuesday, the 1st of August, and as was anticipated, had a very small attendance. The place is only reached by one railroad, and that a long one, almost one hundred miles before making any other railroad connections with the outside world. The place is a pleasant one in many respects, but a very unfortunate one for such a meeting. The meeting was perhaps as good in the work accomplished as could have been expected, but it fell far short of what should have been done. It followed in the old beaten path of former meetings; considering and discussing, practical and theoretical details that ought for the most part to be learned during a good pupilage, or at most, in a thorough college course. There seemed to be an utter aversion to taking up and considering the great principles that underlie the progress and development of the profession as a whole. The education of those who are yet to enter the profession: The future development and education of those who are now in it, by the various means at the command of such a body, such as the encouraging and fostering local associations, the introduction of lectures and clinics for the profession: Securing definite legal recognition of the profession, such as shall encourage and stimulate the good and honest, and restrain the bad, and protect the public against imposition and abuse. On these subjects and others that might be named, this association ought to give an utterance that would make its impress upon the profession everywhere.

These weighty matters should be attended to, and the others not neglected. This body ought to be, and is for the most part, made up of the representative men of the profession, and their time ought not to be frittered away while in council assembled, upon minor things that should be confined to local societies; such as clinics in minor operations, the exhibition of instruments and appliances; and most decidedly objectionable is it for members of the profession, and particularly members of the body, to bring instruments and appliances for exhibition and sale, and thereby call off the attention of members from business that legitimately belongs to the association. To such an extent has this been the case at the last few meetings, that it was almost impossible to get any committee, however important, together, and retain them long enough to do any business; so that nearly all the business is done in a slipshod, and imperfect manner, and some committees wholly neglect or disregard their work. And especially are lion baths to be discarded hereafter.

Now we hope that the American Dental Association, will arouse itself to the dignity and importance of its position, and go promptly forward in the performance of the work that it has before it, and is so urgently demanded by the profession. T.

